# Molecular identification of diarrheagenic *Escherichia coli* pathotypes and their antibiotic resistance patterns among diarrheic children and in contact calves in Bahir Dar city, Northwest Ethiopia

**DOI:** 10.1371/journal.pone.0275229

**Published:** 2022-09-28

**Authors:** Mequanint Addisu Belete, Tiliksew Bialfew Demlie, Wagaw Sendeku Chekole, Tesfaye Sisay Tessema

**Affiliations:** 1 Department of Veterinary Laboratory Technology, College of Agriculture and Natural Resource, Debre Markos University, Debre Markos, Ethiopia; 2 School of Animal Science and Veterinary Medicine, College of Agriculture and Environmental Sciences, Bahir Dar University, Bahir Dar, Ethiopia; 3 Institute of Biotechnology, University of Gondar, Gondar, Ethiopia; 4 Institute of Biotechnology, Addis Ababa University, Addis Ababa, Ethiopia; CINVESTAV-IPN, MEXICO

## Abstract

Diarrheagenic *Escherichia coli* strains are an essential cause of diarrheal infection in younger children and animals. The study was focused on understanding the associated characteristics of various DEC strains among children and calves, establishing the possible zoonotic transmission, and determining their antibiotic resistance patterns. Samples from 144 acute diarrheic children and 50 diarrheic calves were collected and processed using traditional culture methods. The molecular identification of pathotypes was completed using primer-specific polymerase chain reaction (PCR) targeting ten virulence genes (*stx1*, *stx2*, *eae*, *aatA*, *lt*, *st*, *ial*, *hlyA bfpA*, *and daaE*) related to six DEC pathotypes (EPEC, ETEC, EHEC, EAEC EIEC, and DAEC). The antimicrobial susceptibility testing was carried out using the Kirby-Bauer disk diffusion method. Colonies from 74 study subjects (54 diarrheic children and 20 diarrheic calves) were positive for *E*. *coli* isolates. Subsequent PCR detection discovered that 77% of children and 85% of calves’ isolates were positive for one or more virulence genes typical of particular strains. Among those ETEC [(18%), (26%)] is being the maximum predominant, and [(15%), (15%)] were positive for STEC, [(13%), (8%)] for atypical EPEC, [(6%), (7%)] for EHEC, [(6%), (5%)] for EAEC, and [(6%), (4%)] for EIEC strains in children’s and calves, respectively. Of the identified *E*. *coli* isolates, about 29% were found to be hybrid isolates. ETEC (66.7%) and STEC (58.9%) strains showed a better detection rate in contact children with diarrheic calves than children with no contacts. Most antibiotic resistances were obtained towards amoxicillin (64.9%), gentamycin (56.8%), and ampicillin (54.1%). Up to sixty-five percent of isolates were resistant to a minimum of three categories of antibiotics. This is the primary report on the wide occurrence of the six-diarrheagenic *Escherichia coli* strains, and ETEC was found to be the predominant pathotype among children and contact calves in Ethiopia.

## Introduction

Bacterial diarrheal diseases are one of the important reasons for mortality and morbidity in younger children and animals, especially in low and middle-income countries (LMICs) [1, 2]. Many diarrheagenic enteric pathogens are zoonotic and transmitted from animals to human beings via many pathways. Current epidemics have highlighted direct or indirect contact with an animal reservoir as some other major path of transmission for those enteric pathogens [3, 4].

A range of known microorganisms inclusive of bacteria, viruses, and parasites could be the etiological agents of diarrhea. Globally, *E*. *coli* is one of the common causes of infectious diarrhea [5]. The relationship between *E*. *coli* and the host is described as commensalism because the *E*. *coli* microbiota presents a few benefits to its host [6, 7]. However, in a selected situation, highly adapted *E*. *coli* strains can cause infections such as Urinary Tract Infection (UTI), diarrhea, and bloodstream infections, among others [8]. The natural reservoirs of pathogenic *E*. *coli* are the intestinal tracts of animals, specifically ruminants [9].

Identification of bacterial techniques available in most developing countries for DEC strains is a traditional bacterial culture and biochemical test that’s insufficient since those strains can’t be simply distinguishable from the normal fecal flora [10]. Polymerase chain reaction (PCR) has emerged as one of the leading molecular techniques to detect genes encoding virulence elements in DEC isolates and permit the rapid, precise detection of distinct pathotypes of DEC [11].

Currently, based on the presence of defined virulence factors, their epidemiology, and medical manifestations of the disease, diarrheagenic *E*. *coli* strains are categorized into six pathotypes: enterotoxigenic *E*. *coli* (ETEC), enteropathogenic *E*. *coli* (EPEC), enterohemorrhagic *E*. *col* (EHEC) or Shiga toxin-producing *E*. *coli* (STEC), enteroaggregative *E*. *coli* (EAEC), enteroinvasive *E*. *coli* (EIEC), and diffuse-adhering *E*. *coli* (DAEC) [12–14].

The growing prevalence of antibiotic-resistant microorganisms in both human beings and animals requires urgent attention and accurate and rapid diagnostic methods. Transmission of resistant *E*. *coli* may additionally arise from animal reservoirs via feces and transfer antimicrobial resistance traits, either directly or via the food chain [15].

Several nearby studies were performed to observe the prevalence and determinants of enteropathogens causing diarrhea in Ethiopia [16–21]. Similarly, there is also research on *E*. *coli* among under-five children and diarrheic calves within the country [22–27]. However, a complete file regarding the pathogenic strain of *E*. *coli*-mediated diarrhea is sparse, and inclusive molecular research is not available in the study area. Additionally, there was no preceding research performed from the One Health perspective. The study was focused on understanding the associated characteristics of various DEC strains among children and calves, establishing the possible zoonotic transmission, and determining their antimicrobial sensitivity patterns in Bahir Dar city, Northwest Ethiopia.

This study targeted children under the age of five years and calves dwelling nearby. We tend to hypothesize this close association may cause high rates of pathogenic *E*. *coli* transmission from calves to children and vice versa.

## Material and methods

### Ethics approval

The institutional Ethical and Environmental Considerations review committee of Bahir Dar University approved the study (REF: BU/144/1.3.4/20). Written consent was received from parents/guardians of the children once the intention and the character of the study were outlined to them.

### Study area

The study was done at selected health institutions (Han health center, Shimpt health center, Addis Alem primary hospital, and Felege Hiwot comprehensive specialized hospital), farms, and homesteads of diarrheic children in Bahir Dar city, which is the capital city of Amhara regional state, Northwest, Ethiopia. It is placed approximately 565 km away from, Addis Ababa, between latitude and line of longitude of 11 36’-12 15’N and 37 23’-38 20’E, respectively, and has a mean elevation of 180 m above the sea level [28].

Bahir Dar is a densely populated city that has several urban and peri-urban animal husbandry practices that function as a number one supply of income. These production structures in particular evolved in inadequate agricultural land because of urbanization. In those situations, the presence of livestock animals close to humans and exposure to the animal’s feces is more common.

### Study design, population, and settings

A cross-sectional study was carried out from December 2020 to June 2021. Stool and fecal samples were collected using purposive sampling methods. Participants were children under five years of age with acute diarrhea and hospitalized or visited the institutions as an outpatient.

Animals included during this study were calves below four months of age, clinically affected with diarrhea, and residing in close contact with children. All calves showing diarrhea were samples regardless of different clinical parameters taken into thought. Each household was taken into consideration just once throughout the study period. Samples from households having more than one diarrheic calves were pooled together.

### Questionnaire survey

A structured questionnaire was used and completed by skilled nurses and laboratory technicians working in each respective health center and hospital with a face-to-face interview. It was to assess the information regarding gender, age, and clinical symptoms of the children from the parents/guardians and for enrollment of caregivers who reported the presence of diarrheal calves and children having contact with diarrheic calves.

### Samples collection and transportation

Stool samples were collected from diarrheic children who fulfilled the inclusion standards and placed in a sterile clean, leak-proof plastic container onto which buffered peptone water was added for enrichment.

Following enrollment of caregivers, who stated the presence of diarrheal calves at the child’s compound employing a standard questionnaire, the patients were accompanied to their houses at the addresses provided by consenting parents. Once every animal became restrained, about 10 ml of diarrheic feces were gathered directly from the rectum using disposable plastic gloves [29]. Specimens were placed in a dry, leak-proof, sterile plastic tube, and raw information was continually recorded (date, calf id, sex, age, breed).

Then the samples were transported in an icebox containing an ice pack within 24 hours of collection to the Amhara Public Health Institute (APHI), where specimens were stored at refrigeration temperature (+4°C) until processed. Fecal and stool samples were processed within 24–48 hr. after collection.

### Isolation and identification of *E*. *coli*

For isolation of *E*. *coli*, MacConkey agar (MCA) (Himedia) and Eosin Methylene Blue agar (EMB) (Himedia) were used as differential and selective plating media. Each fecal sample was streaked onto Eosin methylene blue (EMB) agar using a wire loop in a laminar flow hood. Following overnight incubation, three colonies in each sample displaying greenish metallic sheen characteristics on EMB agar were picked up and taken into consideration as presumptive *E*. *coli*. Additionally, four to five colonies were then picked up and streaked on MacConkey agar and incubated at 37°C for 24 hours for pink colonies (lactose fermenting) and pale colonies (non-lactose fermenting) characteristics. Three well-separated colonies were cultured on nutrient agar and taken as a pure *E*. *coli* culture. Then each culture was subjected to a series of standard biochemical tests, IMViC (indole, methyl red, Voges-Proskauer, citrate). Identification of suspected *E*. *coli* colonies was performed following standard diagnostic microbiologic methods described in [30]. All cultures ferment lactose within 24hrs., and deliver IMViC patterns of + +—-; were considered to be *E*. *coli* and stored at -20°C in Tryptone Soya broth containing 25% glycerol for further molecular analysis.

### Molecular detection of virulence genes of diarrheagenic *E*. *coli*

To extract DNA, a single bacterial colony grown on EMB agar was inoculated into the Tryptone Soya broth and incubated at 37°C for 18–24 hours. After incubation, an aliquot of 2 ml was taken from Tryptone Soya broth and transferred into sterile Eppendorf tubes. Each bacterial suspension was centrifuged at 13, 000 rpm for five min at 4°C. The supernatant was discarded, and the pellet was suspended in 200 μl of sterile distilled water. The cell suspension in an Eppendorf was then boiled for 10 min. in a heat block at 95°C and immediately cooled at -20°C for at least 30 min. before centrifugation at 13,000 rpm for 5 min at 4°C. The supernatant containing DNA was transferred into a new Eppendorf tube, and it was used directly as template DNA for PCR amplification [31]. The quality and quantity of the extracted DNA were assessed using 1.5% agarose gel electrophoresis and Nanodrop 2000/2000C Spectrophotometer (Thermo Scientific™, USA).

Conventional PCR assays were performed using 10 specific primers targeting different virulence genes as it was previously described [32–40] (S1 Table). Each PCR test was accomplished in a final 25μl reaction containing 2.5 μl of PCR buffer with 7.5mM MgCl_2_ (10X, Himedia), 1μl of dNTP (100Mm, Himedia), 0.5 μl of each forward and reverses primers (Bioneer), 0.5 μl of Taq DNA polymerase (5U/μl, Solis BioDyne), 3 μl of a template. Volume adjustment was performed by adding 17 μl of nuclease-free water. Samples carrying the relevant virulence gene (s) taken from previous works and nuclease-free water were used as positive and negative controls, respectively.

All amplifications were carried out in Prima 96 plus Thermal Cycler (Himedia Laboratories, India). Cycling conditions consisted of initial denaturation at 95°C or 3 min., followed by 35 cycles of amplification (denaturation, annealing, and extension temperature with time presented in S1 File) and final extension at 72°C for 10 min. PCR products were submitted to electrophoresis in 1.5% (w/v) agarose gel (Bio Basic Inc. Canada) stained with (10mg/ml) ethidium bromide. The electrophoresis was carried out at 100 V for 45 min in 1X TAE buffer (40 mM Tris, 1 mM EDTA, and 20 mM glacial acetic acid, pH 8.0). A 100-bp DNA molecular weight marker (Solis BioDyne, Estonia) was used to estimate the product size. The resulting band’s patterns were visualized and recorded using a gel documentation system (BioRAD, USA).

### Antimicrobial susceptibility tests

The antimicrobial susceptibility test of the DEC isolates was determined by standard Kirby Bauer’s disk diffusion method against Ampicillin (AMP), 10μg, Amoxicillin (AMX),10μg, Chloramphenicol (C), 30μg, Ciprofloxacin(CIP), 5 μg, Gentamycin (GEN),10μg, Norfloxacin (NX), 10μg, Compound sulphonamides (S3), 300 μg, Tetracycline (TE), 30μg, Trimethoprim (W5), 5 μg (Thermo Scientific™ Oxoid™, United Kingdom).

From pure overnight culture, 4–6 bacterial colonies were suspended in 5ml nutrient broth (Himedia Laboratories, India) and incubated for 24 hours at 37°C. The turbidity of the broth culture was equilibrated to match 0.5 McFarland standards. The surface of the Mueller-Hinton agar plate (Himedia Laboratories, India) was evenly inoculated with the culture using a sterile cotton swab. The antibiotic discs were placed onto the surface of the inoculated Muller-Hinton agar. After 18–24 hours of growth, the diameters of inhibition around the discs were measured, recorded, and interpreted as sensitive, intermediate, or resistant according to clinical and laboratory standards institute CLSI [41]. *E*. *coli ATCC 25922* was used as a control in all assays. *E*. *coli* isolates resistant to three or additional categories of antibiotics were considered multi-drug resistant (MDR).

### Data analysis

Data on risk factors, clinical characteristics, the detection rate of *E*. *coli*, and the abundance of the identified pathogenic strain were summarized using descriptive statistics. Data on antibiotic susceptibility profiles were presented in tables and bar graphs. The possible association between risk factors, clinical characteristics, and the presence of specific virulence genes were assessed using chi-square tests. The analyses were performed using statistical software, SPSS version 23 (IBM, Colorado, USA), and differences among variables were considered statistically significant to the extent of the p-value of < 0.05.

## Results

A total of 194 samples consisting of 144 stool samples from children and 50 fecal samples from calves were enclosed during this study. Out of the overall samples, presumptive *E*. *coli* isolates were identified from 74 (38.3%) of the samples following morphological features on MacConkey agar, EMB agar, and their IMViC biochemical tests (+ +—-). The positivity constituted 54 (37.8%) samples from diarrheic children and 20 (40%) samples from diarrheic calves.

Amongst children included in the present study, 78 (54.1%) of them were below 24 months, 46 (31.9%) were between 24 and 47 months, and 20 (13.8%) were over 48 months (Table 1). About 80 (55.5%) of them were male, and 64 (44.4%) were female. The age distribution of the isolation rate of *E*. *coli* was significant (p = 0.013), while the occurrence of *E*. *coli* according to the sex of subjects was not statistically significant (p = 0.874). Among children, 44.4% were fed a mixture of breast milk and formulation milk, 34.7% were fed with solids and 20.8% were only allowed to suckle the breast milk. The isolation rate of *E*. *coli* was significantly corresponding with the feeding types (p = 0.008) (Table 1).

**Table 1:** Occurrence of *E*. *coli* based on age, sex, and clinical features in diarrheic children.

Variables	Categories	No. of Positive (%)	No. of Negative (%)	p-value
Sex	Female	24(44.4)	40(44.4)	0.874
	Male	30(55.6)	50(55.6)	
Age	0–5 months	1(1.9)	9(10)	0.013*
	6–11 months	9(16.7)	20(22.2)	
	12–23 months	13(24.1)	26(28.9)	
	24–35 months	18(33.3)	13(14.4)	
	36–47 months	7(13)	8(8.9)	
	48–59 months	6(11.1)	14(15.6)	
Feeding type	Breast milk	8(14.8)	22(24.4)	0.008*
	Breast milk+	23(42.5)	41(45.5)	
	Solid feed	23(42.5)	27(30)	
Diarrhea type	Watery	22(40.7)	36(40)	0.817
	Mucoid	7(12.9)	13(14.4)	
	Bloody	15(27.7)	18(20)	
	Loose	9(16.6)	23(25.5)	

No.: Number

* = P ≤ 0.05

A better occurrence of *E*. *coli* inflicting diarrhea was detected in calves older than 4 weeks (28%), exotic breeds (60%), and females (60%). Both sex and breed showed significant differences in the prevalence of *E*. *coli* (p = 0.034), (p = 0.024), respectively whereas, the age of calves had no significant difference. Samples positive for *E*. *coli* are grouped based on age, breed, and sex as presented (Table 2).

**Table 2:** Occurrence of *E*. *coli* based on age, sex, and breed of diarrheic calves.

Variables	Categories	No. of Positive (%)	No. of Negative (%)	p-value
Sex	Female	12(60)	15(60)	0.034*
	Male	8(40)	10(40)	
Age	<1weeks	2(10)	4(16)	0.404
	1–4 weeks	6(30)	7(28)	
	4–6 weeks	5(25)	9(36)	
	6–12 weeks	9(45)	5(25)	
Breed	Local	3(15)	6(24)	0.024*
	Cross	5(25)	7(28)	
	Exotic	12(60)	12(48)	

No.: Number

* = P ≤ 0.05

All phenotypically presumptive isolates were further screened through PCR for the presence of 10 specific virulence genes *stx1*, *stx2*, *eae*, *aatA*, *lt st*, *ial*, *and daaE*, *hlyA*, and *bfpA*. Among the overall 74 samples, 59 (79.7%) samples were positive for at least one of the targeted virulence genes of *E*. *coli* (Table 3).

**Table 3:** Distribution of virulence genes in *E*. *coli* isolated from diarrheic children and calves.

Virulence gene	No. of samples from children (%)	No. of samples from calves (%)	Total (%)
*Eae*	15(27.8)	3(15)	18(24.3)
*stx1*	14(25.9)	3(15)	17(22.9)
*stx2*	11(20.4)	5(25)	16(21.6)
*ehlyA*	7(13.0)	2(10)	9(12.1)
*aatA*	6(11.1)	2(10)	8(10.8)
*Ial*	8(14.8)	1(5)	9(12.1)
*Lt*	13(24.1)	4(20)	17(22.9)
*St*	16(29.6)	10(50)	26(35.1)

*eae*, attaching effacing gene; *stx1*, Shiga-like toxin I gene; *stx2*, Shiga-like toxin II gene; *ehlyA*, *E*. *coli* hemolysin gene; *bfpA*, bundle forming pili gene; aatA, aggregative adherence gene; *ial*, invasive gene; *lt*, Heat liable toxin gene; *st*, Heat stable toxin gene

Out of 54 *E*. *coli*, positive samples from diarrheic children, 42 (77.7%) revealed the detection of either of the virulence genes (Table 3). The maximum and minimum frequent virulence genes were the *st* (29.6%), and *aatA* (11.1%), respectively. However, none of the samples were positive for *daaE* and *bfpA* genes.

Among 20 *E*. *coli* positive samples from diarrheic calves, 17 (85%) proved the existence of at least one of the targeted virulence genes (Table 3). The most common gene was the *st* gene similar to the children group accounting for 50% of all cases. Only 1 (5%) sample was positive for the *ial* gene. In contrast, 15% of *E*. *coli* isolates were found non-pathogenic, based on the absence of all investigated genes.

**Fig 1:**
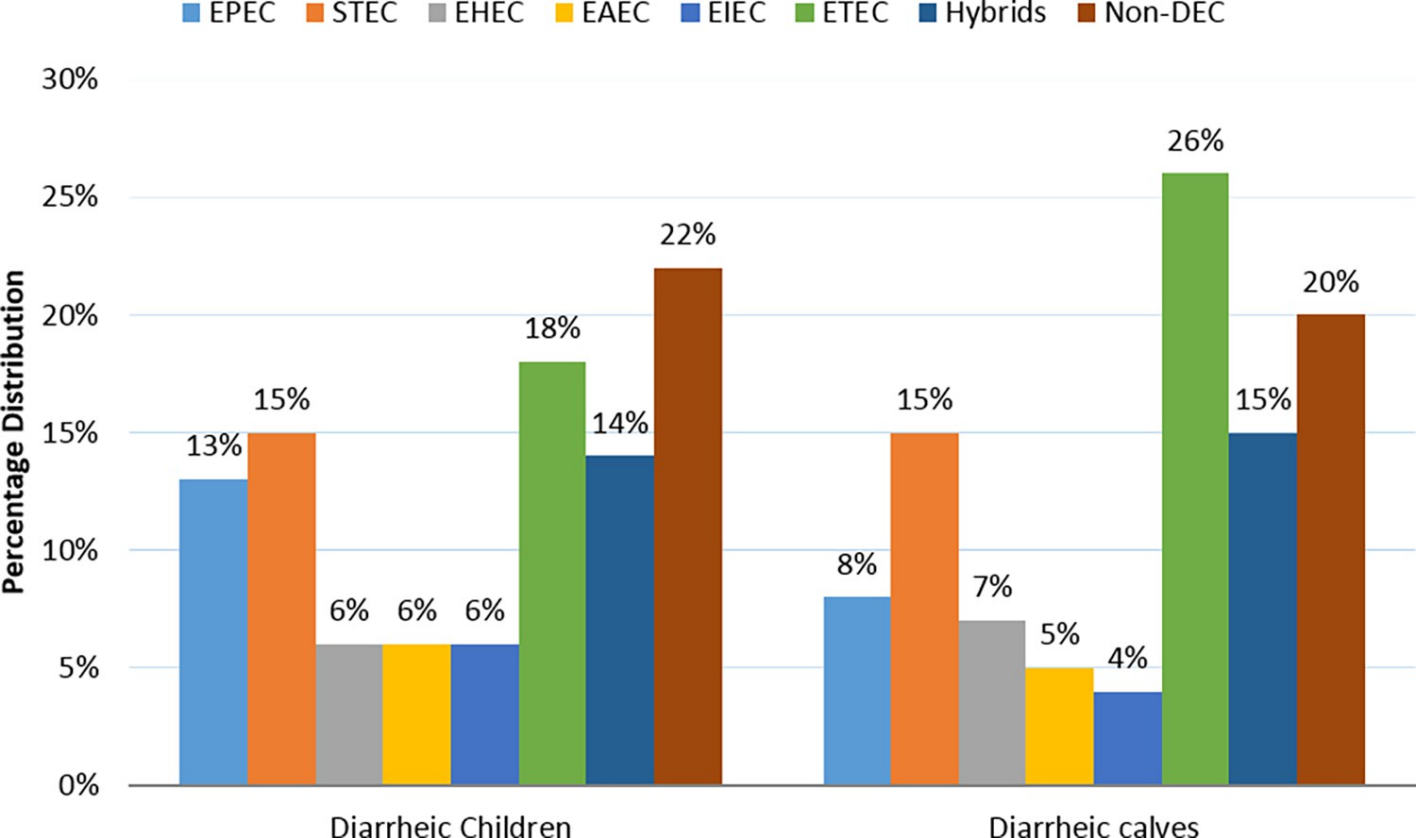
Distribution of DEC pathotypes among diarrheic children and claves.

DEC strains were determined as follows: the presence of *bfpA* and *eae* gene for typical EPEC, the presence of *eae* gene only for atypical EPEC, and the presence of *eae*, *stx1*, *stx2*, *ehlyA* gene for EHEC. In addition, the presence of *stx1* and or *stx2* gene for STEC, the presence of *lt* and or the *st* gene for ETEC, the presence of *aatA* gene for EAEC, and the presence of *ial* gene for EIEC. Isolates possessing two or more virulence genes from different *E*. *coli* strains were identified as a hybrid. Based on these criteria and the virulence gene profile, the 74 samples were categorized as follows: 36 samples harbored either *lt* or *st* or both genes and were grouped as ETEC strains, while eight isolates were positive for EAEC strains. Samples negative to all virulence genes accounted for 15 (20%) of the total samples (Fig 1).

Detection of DEC pathotypes was more frequent in isolates from males (53.2%) than females (46%) (Table 4). The age wise distributions of DEC pathotypes were found to be inconsistent. EPEC strain was the only pathotype detected in the 0–5 months age groups (Table 4). Five DEC categories (ETEC, EAEC, EHEC, STEC, and EIEC) were found inconsistent abundance in all age groups except for children aged 0–5 months (Table 4). Regarding the patient’s clinical symptoms, watery diarrhea (40.2%) was the most common diarrheal consistency, of which in the study subjects, 29.7% of ETEC strains were detected (Table 4). Concerning the feed types, 42% and 41% of DEC pathotypes were detected from solid and combined breast and formula milk, respectively (Table 4).

**Table 4:** Distribution of DEC pathotypes with clinical features and risk factors in diarrheic children.

Pathotype with respective gene	Variables														
	Sex		Age (months)						Type of Diarrhea				Feed type		
	M	F	0–5	6–11	12–23	24–35	36–47	48–60	W	B	M	L	B	B&F	S
EPEC total *(N = 15)*	8	7	1	2	4	5	2	1	6	2	4	3	3	6	6
atypical EPEC *eae*	8	7	1	2	4	5	2	1	6	2	4	3	3	6	6
STEC total *(N = 17)*	9	8	-	3	5	6	1	2	6	2	6	3	5	7	5
*stx1* only	3	3	-	1	2	2	0	1	2	1	2	1	3	3	0
*stx2* only	2	1	-	1	1	1	0	0	1	0	1	1	0	1	2
*stx1*and *stx2*	4	4	-	1	2	3	1	1	3	1	3	1	2	3	3
EHEC total *(N = 7)*	4	3	-	1	2	2	1	1	3	1	1	2	1	3	3
*eae*, *stx1*, *ehlyA*	1	1	-	-	1	1	-	-	1	-	1	-	-	1	1
*eae*,*stx1*,*stx2*,*ehlyA*	3	2	-	1	1	1	1	1	2	1	1	1	1	2	2
EAEC total *(N = 6)*	3	3	-	1	1	2	1	1	3	1	1	1	-	3	3
*aatA*	3	3	-	1	1	2	1	1	3	-	2	1	-	3	3
EIEC total *(N = 8)*	4	4	-	1	3	2	1	1	3	1	2	2	1	3	3
*ial*	4	4	-	1	3	2	1	1	3	1	2	2	1	3	3
ETEC total *(N = 24)*	13	11	-	4	7	8	3	2	11	4	6	3	3	10	11
*lt* only	5	3	-	1	3	2	1	1	3	1	3	1	1	4	3
*st* only	6	6	-	2	3	4	2	1	5	2	3	2	2	5	5
*st* and *lt*	2	2	-	1	1	1	1	-	2	1	1	-	-	2	2
Hybrid total*(N = 15)*	8	7	-	2	4	5	2	2	5	3	4	3	2	6	7
ETEC/EIEC	2	2	-	1	1	1	1	-	1	1	1	1	1	1	2
*st + ial*	2	2	-	1	1	1	1	-	1	1	1	1	1	1	2
ETEC/EAEC	2	1	-	1	1	1	-	-	1	-	1	1	-	2	1
*st + aatA*	2	1	-	1	1	1	-	-	1	-	1	1	-	2	1
STEC/ ETEC	4	4	-	-	2	3	1	2	3	2	2	1	1	3	4
*st+stx2*	1	1	-	-	1	1	-	-	1	-	1	-	-	1	1
*stx1+lt+st*	1	1	-	-	-	2	-	-	1	1	-	-	-	-	2
*stx1+stx2+st*	2	2	-	-	1	-	1	2	1	1	1	1	1	2	1
Total *(N = 92)*	49	43	1	14	26	30	11	10	34	14	24	17	15	38	38

W = watery; B = bloody; M = mucoid; L = loose B = breast feed only; B&F = breast and formula milk; S = solid food

The occurrence of each virulence gene, and pathogroups of diarrheic calves, are shown in Table 5. In this study, the distributions of DEC pathotypes were minor between the male and female diarrheic calves. The *st* and *lt* genes were detected across all age groups except the 4–6 weeks age groups. Among the pathotypes, STEC and ETEC strains were found only in calves aged > 1 week. Relative to the breed of calves, DEC pathotypes were most commonly detected in exotic breeds. Of pathotypes, ETEC was the most frequently detected pathotype (26%) followed by equal 17% detections of STEC and hybrid pathotypes.

**Table 5:** Distribution of DEC pathotypes with clinical features and risk factors in diarrheic calves.

Pathotype with respective gene	Variables									
	Sex		Age(weeks)					Breed		
	F	M	<1	1–4		4–6	6–12	Local	cross	Exotic
EPEC total *(N = 3)*	1	2	-		1	-	2	-	-	3
atypical EPEC *eae*	1	2	-	1		-	1	-	-	3
STEC total *(N = 7)*	3	4	`1	2		1	3	1	1	5
*stx1* only	-	1	-	-		-	1	-	-	1
*stx2* only	2	1	-	1		2	-	1	-	2
*stx1*and *stx2*	1	2	1	-		-	2	-	1	2
EHEC total *(N = 3)*	1	2	-	1		-	2	-	-	3
*eae*, *stx1*	-	1	-	-		-	1	-	-	1
*eae*, *stx1*, *ehlyA*	1	-	-	1		-	-	-	-	1
*eae*,*stx1*,*stx2*,*ehlyA*	-	1	-	-		-	1	-	-	1
EAEC total *(N = 2)*	1	1	-	1		1	-	1	-	1
*aatA*	1	1	-	1		1	-	-	-	1
EIEC total *(N =* 1)	1	-	-	-		1	-	-	-	1
*ial*	1	-	-	-		1	-	-	-	1
ETEC total *(N = 12)*	6	6	1	2		1	8	2	5	5
*lt* only	0	2	-	-		-	2	-	-	2
*st* only	4	4	1	2		-	5	1	3	4
*st* and *lt*	2	-	-	1		-	1	1	1	-
Hybrid total *(N = 4)*	2	2	-	2		-	2	-	2	2
STEC/ETEC	1	1	-	1		-	1	-	1	1
*stx1+stx2+st*	1	1	-	1		-	1	-	1	1
EPEC/ETEC	1	1	-	1		-	1	-	1	1
*eae + st*	1	1	-	1		-	1	-	1	1
Total *(N = 32)*	14	17	2	9		4	17	4	8	20

About 50 (34.7%) of the children had direct contact with diarrheic calves. Based on the social history of the children they were lived on farms and exposed to animal manure-contaminated environments. The present study revealed that EIEC (75%), hybrid (60%), EHEC (57.1%), and EPEC (53.5%) strains were shown to have better detection rates in children who had no contact with diarrheic calves compared to the ones exposed. However, ETEC (66.7%) and STEC (58.9%) strains were shown better detection rates in children who have contact with diarrheic calves. EAEC strains accounted for an equal detection of 50% between exposed and non-exposed children to the diarrheic calves.

**Fig 2:**
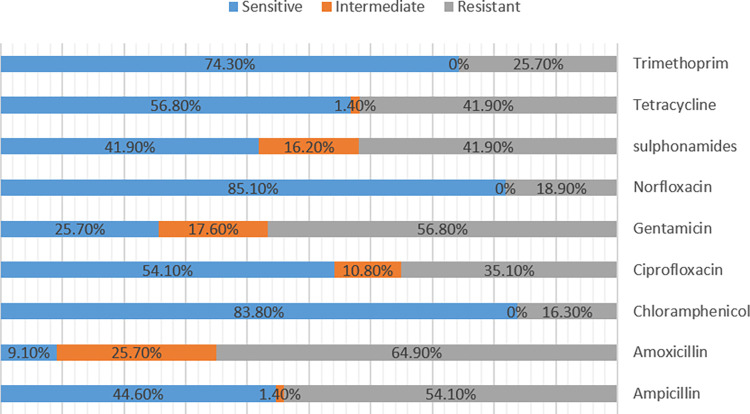
Bar diagram showing antibiotic sensitivity pattern of *E*. *coli* isolates.

A total of 74 DEC isolates from children and calves were further subjected to an antibiotic sensitivity test. The overall susceptibility patterns of *E*. *coli* isolates from diarrheal samples are displayed in Fig 2, Table 6.

**Table 6:** Antimicrobial susceptibility profile of *Escherichia coli* isolates from different sources.

Antibiotics	Interpretations					
	Children (N = 54)			Calves (N = 20)		
	S (%)	I (%)	R (%)	S (%)	I (%)	R (%)
Ampicillin (AMP), 10μg	21(38.9)	1(1.9)	32(59.3)	12(60)	-	8(40)
Amoxicillin (AMX),10μg	-	15(27.8)	39(72.2)	7(35)	4(20)	9(45)
Chloramphenicol(C),30μg	46(85.3)	-	8(14.8)	16(80)	-	4(20)
Ciprofloxacin(CIP), 5 μg	39(72.2)	-	15(27.8)	1(5)	8(40)	11(55)
Gentamycin (GEN),10μg	19(33.3)	20(18.5)	26(48.1)	1(5)	3(15)	16(80)
Norfloxacin (NX), 10μg	50(92.6)	-	4(7.4)	13(65)	4(20)	9(45)
Sulphonamides (S3),300μg	15(27.8)	11(20.4)	28(51.9)	16(80)	1(5)	3(15)
Tetracycline (TE), 30μg	30(55.6)	1(1.9)	23(42.56)	12(60)	-	8(40)
Trimethoprim(W5),5 μg	37(68.5)	-	17(31.5)	18(90)	-	(2)

S = Susceptible, I = Intermediate, R = Resistant

Results indicated that norfloxacin was found the most effective drug with 85.1% sensitivity followed by chloramphenicol 83.8%. The maximum resistance was observed against amoxicillin at 64.9%, followed by gentamycin at 56.8% and ampicillin at 54.1%. When analyzed by source, *E*. *coli* isolates from diarrheic children have shown a higher degree of resistance than isolates from diarrheic calves (Table 6).

Multidrug-resistance (MDR) as a minimum of three antibiotic classes was detected in 48 out of 74 (64.8%) positive isolates. *E*. *coli* isolates resistant to four types of antibiotics 12 (25%), and 3 types of antibiotics 11 (22.9%) were the most common multiple drug resistances. Ten (20.8%), six (12.5%), and four (8.3%) isolates were resistant to six, five, seven, and nine types of antibiotics, respectively. Only one (2.1%) isolate was resistant to eight kinds of antibiotics. The antimicrobial resistance profiles discriminated by DEC pathotypes of children and calves were displayed in the S2 Table.

## Discussion

In the present study, from the total of diarrheic children, 37.8% of them were positive for *E*. *coli* using culture techniques. These findings were nearly similar to those reported by [42], 37.5% and [43], 32.5% from stool samples under five diarrheic children in India and Bangladesh, respectively. However, the current finding was lower than those obtained by other investigators, such as 60.9% found in Burkina Faso [44], 66.9% found in Mozambique [45], and 75% found in Iran [46]. In contrast, the isolation rates of *E*. *coli* were comparatively higher than those reported by [47], 14.2%, and [48], 27.7%. The observed contrasts between the present study and previous reports could be due to variations in sample size, culturing techniques, culture media, and specific incubation conditions.

In total, the presence of *E*. *coli* causing diarrhea among calves in the study area was 40%. The present finding was supported by the works of [29], who reported a 36.8% detection rate of *E*. *coli* in Ethiopia. Moreover, similar results were reported by [49] (44%) and [50] (45.8%) in Bangladesh. However, our isolation rate was proportionally lower than the recent works of [23] and [1], which stated 70.7% and 76.45% prevalence of *E*. *coli* before molecular analysis. The probable reason behind this variation might be associated with differences in the culture procedures and breeds of the studied calves.

The proportion of *E*. *coli* isolates with minimal virulence genes to relevant DEC pathotypes in children was 77.7%, similar to [51], which obtained a detection rate of 76.7% from 215 diarrheic cases in Nigeria. This shows the existence of *E*. *coli* isolates carrying a high level of virulence genes in the study areas. However, reports from other LMICs showed a variation of 6–28% in the detection of DEC isolates [52–54]. This indicates the considerable influences of regional and socio-demographic variations on the distribution of DECs. Moreover, these inconsistencies could be from DNA preparations and PCR conditions used in the above-mentioned studies.

The current finding revealed that *st* was the predominant gene identified in 29.6% of the isolates. This finding is supported by the previous studies of [55] (47.9%) in Bolivia, and [56] (63.1%) in Santiago, Chile. However, the result of this study contradicts other findings reported by [57, 58] with a 9% and 16% occurrence rate of this gene in Peru and Colombia, respectively.

The present study identified six classes of DEC pathotypes in children with diarrhea. The most dominant pathotype was ETEC, followed by STEC strain. This result concurs with a previous study conducted in Kenya that mentioned higher detection rates of ETEC strain [59] Previous studies have defined ETEC as the predominant DEC pathotype among children under five years in LMICs [51, 60, 61]. In this study, ETEC was frequently detected in children > 24 months of age which is similar to the findings reported by [48, 57].

The second most common pathotype was STEC, with a 15% detection rate. The present occurrence rate was higher than the preceding studies by [62] 3.4% in India, [2] 0.3% in Mexico, and [43] 1.2% in Bangladesh. EPEC was the third most common DEC pathotype with a frequency of 13%, in agreement with [45, 52]. However, studies in China were shown EPEC as the most commonly detected pathotype [63]. All EPEC strains detected in this study were atypical-EPEC, lacking the *bfp* gene, similar to works done by [48] in Nigeria, [52] in Iran, and [64] in Vietnam. The detection of atypical EPEC in Ethiopia in the present study further indicates the worldwide distribution of this pathotype. But the existing study contradicts the report done by [45], who indicated atypical EPEC was consistently lower than typical EPEC in sub-Saharan countries.

In the present study, EAEC, EHEC, and EIEC detection rates were uniform at a frequency of 6%, contradicting 21–48% frequencies indicated in previous findings [44, 51, 52]. No isolates carried DAEC-specific virulence genes similar to those reported from Nigerian diarrheic children [48]. The detection of hybrid strains in diarrheic children was in agreement with a14.1% of the preceding report [51]. This could be due to the plasticity nature of *E*. *coli*, which allows the accommodating of multiple virulence genes from different pathotypes. Those variations may also indicate the ongoing changes in the distribution of DEC pathotypes across time and regional perspectives.

Overall, 17 (85%) isolates of calves showed the presence of at least one of the targeted virulence genes. Comparable results were previously recorded by [65], where isolates contained a minimum of one of the investigated virulence genes accounted for 70.6% of fecal samples collected from diarrheic cattle and buffalo calves. But this finding was relatively higher than [66], which stated 44.9% occurrence in Minas Gerais, Brazil, and [67] which indicated a 17% detection in Austria.

Likewise, calves have shown a frequent detection of ETEC pathotypes accounting for 26% of the isolates. This finding was comparable to 28.41% of ETEC occurrences by [1]. In contrast, several studies have indicated a lower detection rate of ETEC isolates [65, 68, 69]. The present finding also disagreed with a study conducted in Ethiopia where no ETEC detection was reported from the diarrheic claves [70]. Most ETEC isolates in the present study had a *st* gene known to encode ST toxins commonly carried in the plasmid. This result contradicts the finding of [71], who described that *lt* was the sole virulence gene causing diarrhea in water buffalo calves in Italy. The *lt* gene responsible for encoding heat-labile enterotoxin was obtained in 20% of the isolates.

The second pathotype with excessive detection was STEC (15%), lower than (35.2%) [69] and (37.3%) [71] from fecal samples of diarrheic calves and buffalo calves, respectively. Among known STEC isolates, the presence of the *stx1* gene (15%) was lower compared to the *stx2* gene (25%). In contrast, in other studies, the *stx1* gene was reported as the major virulence gene compared to the *stx2* gene [72, 73]. The increased STEC detection of 58.9% in this study from children who had direct contact with the diarrheic calves, could be the fact that calves serve as reservoirs of STEC and were transmitted through direct contact or contamination of food with their feces [74]. As a result, the presence of STEC strains in calves raises questions regarding the potential zoonotic transmissions from calves to children.

Most EPEC strains are known to have both bundle-forming pilus (*bfpA*) and *eae* genes. However, in this study, the EPEC strains were atypical and only contained the *eae* gene. The EPEC pathotype represented 8% of *E*. *coli* isolates, slightly lower than 12% [75] and 13% [76] detection rates in Bangladesh and Korea, respectively. A decreased frequency of EPEC strain was also reported by [77–79] in Turkey, Vietnam, and Iran respectively.

In the present study of calves, the *aatA* gene, the virulent factor of EAEC strain, and the *ial* gene, the virulent factor of EIEC were detected at a lower frequency of 5% and 4%, correspondingly. The detection rate of hybrid isolates in diarrheic calves was relatively higher than the 4% occurrence reported by [70]. Inconsistencies ascertained between *E*. *coli* pathotypes and their virulence genes may be related to the season, farm size, and the number of animals. Moreover, other sources of the difference are probably because of hygienic status, management practices, variation in sampling, and differences in the detection methods.

Knowledge of the recent local antibiogram profile of pathogens can help to choose the proper treatment options. Overall, excessive resistance to common drugs like amoxicillin (64.9%), gentamycin (56.8%), ampicillin (54.1%), and tetracycline (41.9%) were observed. The findings ought to allocate those antibiotics that are cheap and illegally acquired over the counter without prescription [80]. Similar findings have been observed in other LMICs. For instance, in Iran, DEC isolated from children showed high resistance to ampicillin, and tetracycline [81]. In Iraq, a study on children’s diarrhea showed 100% resistance to amoxicillin [82]. In Egypt, a study on the antibiogram profiles of DEC found 100% resistance to ampicillin [83].

On the other hand, different *E*. *coli* isolates were sensitive to norfloxacin (85.1%), chloramphenicol (83.8%) and trimethoprim (74.3%). The present finding is consistent with the study conducted by [22], who revealed the highest level of sensitivity to ciprofloxacin and norfloxacin. Similarly, [68] found increased sensitivity of *E*. *coli* isolates to norfloxacin.

Recently, the growing multidrug-resistances (MDRs) have been witnessed and are broadly spreading amongst gram-negative bacteria [84]. In our study, the MDR rates of DEC isolates were over (60%), a finding similar to different studies [52, 75]. But higher than a 54.5% by [69] and 39.5% by [84]. However, the present finding of multidrug-resistant (MDR) phenotypes was lower than those reported by [85]. The current multidrug resistance is probably because those antibiotics are widely available and used inappropriately by humans and on animal production farms, or those bacteria are known to accumulate genes responsible for coding antimicrobial resistance mechanisms [86, 87].

## Conclusion

Our research discovered a wide occurrence of DEC strains and the existence of hybrid infection among children and calves in Bahir Dar, Ethiopia. Six DEC strains, particularly ETEC, EPEC, EAEC, EHEC, STEC, and EIEC, were identified in diarrheic children and contact calves, with ETEC being the predominant pathotype. The associated patterns in the identified DEC among diarrheic children and calves could indicate potential zoonotic transmissions of certain pathotypes among the study subjects. Conducting appropriate phylogenetic studies using an inclusive One-Health approach with different experimental settings may provide relevant information concerning the potential zoonotic transmission. Given this finding, for a better understanding of pathogens responsible for diarrhea among children and calves, we strongly recommend the need to apply a case-control study. The present study has revealed the development of an excessive rate of antimicrobial resistance to commonly used antibiotics. Hence, regular antimicrobial susceptibility surveillance programs needed to be implemented to reduce the growing antimicrobial resistance. The results we presented in this study might have an impact on designing control and prevention strategies against child diarrhea particularly caused by DECs.

## Supporting information

S1 Table PCR oligonucleotide sequences, amplicon sizes and PCR conditions used for amplification. (DOCX)

S2 Table Antimicrobial resistance profiles of DEC strains from children and calves. (DOCX)

S1 File Representative Agarose gel electrophoresis raw images of PCR amplified products. (PDF)
