# Supplementary material for: Molecular identification of diarrheagenic Escherichia coli pathotypes and their antibiotic resistance patterns among diarrheic children and in contact calves in Bahir Dar city, Northwest Ethiopia
Source: PLoS One. 2022 Sep 28;17(9):e0275229. doi: 10.1371/journal.pone.0275229 (PMC9518915; doi:10.1371/journal.pone.0275229)
Supplement: S1 Table — (DOCX) [file pone.0275229.s001.docx]

**S1 Table.** **PCR oligonucleotide sequences, target virulence-associated genes, amplicon sizes and PCR conditions used for amplification**

| **Primer code** | **Oligonucleotide Nucleotide sequence 5’ to 3’** | **Target gene** | **Pathogenic *E. coli* strain** | **PCR Conditions** | | | | | | **Product**  **size (Bp)** | **Reference** |
| --- | --- | --- | --- | --- | --- | --- | --- | --- | --- | --- | --- |
|  |  |  |  | **Denaturation** | | **Annealing** | | **Extension** | |  |  |
|  |  |  |  | Temp | Time | Temp | Time | Temp | Time |  |  |
| EAE1  EAE2 | F: AAACAGGTGAAACTGTTGCC  R: CTCTGCAGATTAACCTCTGC | *eae* | EPEC/EHEC | 95 ^0^C | 40 sec | 55 ^0^C | 30 sec | 72 ^0^C | 1 min | 490 | [32] |
| EVS1  EVC2 | F: ATCAGTCGTCACTCACTGGT  R: CTGCTGTCACAGTGACAAA | *stx1* | STEC/EHEC | 95 ^0^C | 40 sec | 55 ^0^C | 30 sec | 72 ^0^C | 1 min | 110 | [33] |
| EVT1  EVT2 | F: CAACACTGGATGATCTCAGC  R: CCCCCTCAACTGCTAATA | *stx2* | STEC/EHEC | 95 ^0^C | 40 sec | 55 ^0^C | 30 sec | 72 ^0^C | 1 min | 350 | [33] |
| EHEC F  EHEC R | F: ACGATGTGGTTTATTCTGGA  R: CTTCACGTCACCATACATAT | *hlyA* | EHEC | 95 ^0^C | 40 sec | 45 ^0^C | 40 sec | 72 ^0^C | 1 min | 167 | [34] |
| EAEC F  EAEC R | F: CTGGCGAAAGACTGTATCAT  R: CAATGTATAGAAATCCGCTGTT | *aatA* | EAEC | 95 ^0^C | 40 sec | 48 ^0^C | 30 sec | 72 ^0^C | 1 min | 630 | [35] |
| BFPF  BFPR | F: AATGGTGCTTGCGCTTGCTGC  R: GCCGCTTTATCCAACCTGGTA | *bfpA* | typical EPEC | 95 ^0^C | 40 sec | 45 ^0^C | 40 sec | 72 ^0^C | 1 min | 324 | [36] |
| ST1  ST2 | F: TTT ATT TCT GTA TTG TCT T  R:GCAGGATTACAACACAATTC | *st* | ETEC | 95 ^0^C | 40 sec | 55 ^0^C | 30 sec | 72 ^0^C | 1 min | 294 | [37] |
| LT1  LT2 | F: GCTCTATGTGCATACCGAGT  R:CCATACTTGATTGCCGCAAT | *lt* | ETEC | 95 ^0^C | 40 sec | 48 ^0^C | 30 sec | 72 ^0^C | 1 min | 696 | [38] |
| IAL F  IAL R | F: CTGGATGGTATGGTGAGG  R: GGAGGCCAACAACATTATTTCC | *ial* | EIEC | 95 ^0^C | 40 sec | 55 ^0^C | 30 sec | 72 ^0^C | 1 min | 320 | [39] |
| daaE 1  daaE 2 | F: GAACGTTGGTTAATGTGGGGT  R:TATTCACCGGTCGGTTATCAG | *daaE* | DAEC | 95 ^0^C | 40 sec | 48 ^0^C | 30 sec | 72 ^0^C | 1 min | 542 | [40] |

TEMP= Temperature, Sec = Second, Min = Minute
