# Supplementary material for: Molecular identification of diarrheagenic Escherichia coli pathotypes and their antibiotic resistance patterns among diarrheic children and in contact calves in Bahir Dar city, Northwest Ethiopia
Source: PLoS One. 2022 Sep 28;17(9):e0275229. doi: 10.1371/journal.pone.0275229 (PMC9518915; doi:10.1371/journal.pone.0275229)
Supplement: S2 Table — (DOCX) [file pone.0275229.s002.docx]

| **Antibiotics** | **EPEC *N (%)*** | | **STEC *N (%)*** | | **EHEC *N (%)*** | | **EAEC *N (%)*** | | **EIEC *N (%)*** | | **ETEC *N (%)*** | | **Hybrid *N (%)*** | |
| --- | --- | --- | --- | --- | --- | --- | --- | --- | --- | --- | --- | --- | --- | --- |
|  | Children  (n=15) | Calf  (n=3) | Children  (n=17) | Calf  (n=7) | Children  (n=7) | Calf  (n=3) | Children  (n=6) | Calf  (n=2) | Children  (n=8) | Calf  (n=1) | Children  (n=24) | Calf  (n=12) | Children  (n=15) | Calf  (n=7) |
| **AMP** | 9(60) | 1(33.3) | 10(58.8) | 3(42.8) | 4(57.1) | 1(33.3) | 4(66.6) | 1(50) | 5(62.5) | - | 14(58.3) | 5(41.6) | 9(60) | 3(42.8) |
| **AMX** | **11(73.3)** | 1(33.3) | 12(70.5) | 3(42.8) | 5(71.4) | 2(66.6) | 4(66.6) | 1(50) | **6(75)** | **1(100)** | 17(70.8) | 5(41.6) | **11(73.3)** | 3(42.8) |
| **C** | 2(13.3) | 1 (33.3) | 3(17.6) | 1(14.2) | 1(14.2) | 1(33.3) | 1(16.6) | - | 1(12.5) | - | 4(16.6) | 2(16.6) | 2(13.3) | 1(14.2) |
| **CIP** | 4(26.6) | 2(66.6) | 5(29.4) | 4(57.1) | 2(28.5) | 2(66.6) | 2(33.3) | 1(50) | 2(25) | **1(100)** | 7(29.1) | 7(58.3) | 4(26.6) | 4(57.1) |
| **GEN** | 7(46.6) | 3(100) | 8(47) | **6(85.7)** | 3(42.8) | 2(66.6) | 3(50) | **2(100)** | 4(50) | 1**(100)** | 12(50) | 10(83.3) | 7(46.6) | 6(85.7) |
| **NX** | 1(6.6) | 2(66.6) | 1(5.8) | 4(57.1) | 1(14.2) | 2(66.6) | - | 1(50) | 1(12.5) | 1**(100)** | 2(8.3) | 6(40) | 1(6.6) | 4(57.1) |
| **S3** | 8(53.3) | 1(33.3) | 9(52.9) | 1(14.2) | 4(57.1) | 1(33.3) | 3(50) | - | 4(50) | - | 12(50) | 2(16.6) | 9(60) | 1(14.2) |
| **TE** | 6(40) | 1(33.3) | 7(41.1) | 3(42.8) | 3(42.8) | 1(33.3) | 3(50) | 1(50) | 4(50) | - | 10(41.6) | 5(41.4) | 6(40) | 3(42.8) |
| **W5** | 5(33.3) | - | 5(29.4) | 1(14.2) | 2(28.5) | - | 2(33.3) | **2(100)** | 3(37.5) | - | 8(33.3) | 1(8.3) | 5(33.3) | 1(14.2) |
| **MDR** | 10(66.6) | 2(66.6) | 11(64.7) | 4(57.1) | **5(71.4)** | 2(66.6) | 4(66.6) | 1(50) | 5(62.5) | **1(100)** | 16(66.6) | 7(58.3) | 10(66.6) | 4(57.1) |

**S2 Table.** **Antimicrobial resistance profiles of DEC strains from children and calves**

AMP *= Ampicillin;* AMX *= Amoxicillin;* C*=* *Chloramphenicol;* CIP*=* *Ciprofloxacin;* GEN*=* *Gentamycin;* *Norfloxacin;* S3= Sulphonamides; TE= *Tetracycline;* W5= *Trimethoprim*; MDR= Multiple drug resistance; Bold indicates the category with major proportion of resistant strain
