# Supplementary figures and images for: Molecular identification of diarrheagenic Escherichia coli pathotypes and their antibiotic resistance patterns among diarrheic children and in contact calves in Bahir Dar city, Northwest Ethiopia
Source: PLoS One. 2022 Sep 28;17(9):e0275229. doi: 10.1371/journal.pone.0275229 (PMC9518915; doi:10.1371/journal.pone.0275229)

M +C 1 2 3 4 5 -C

449bp(eae)

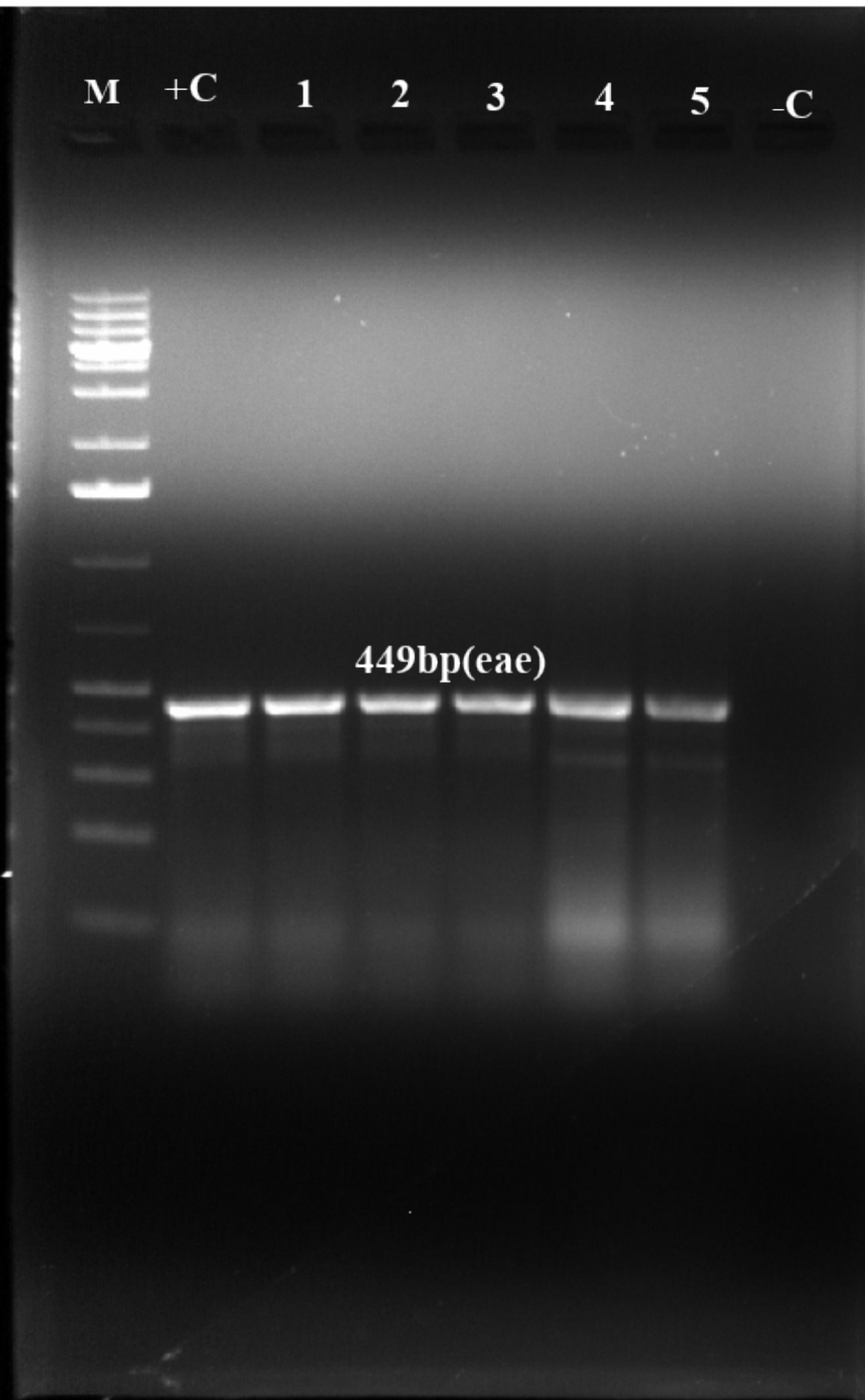

M +C 1 2 3 -C

110bp(stx1)

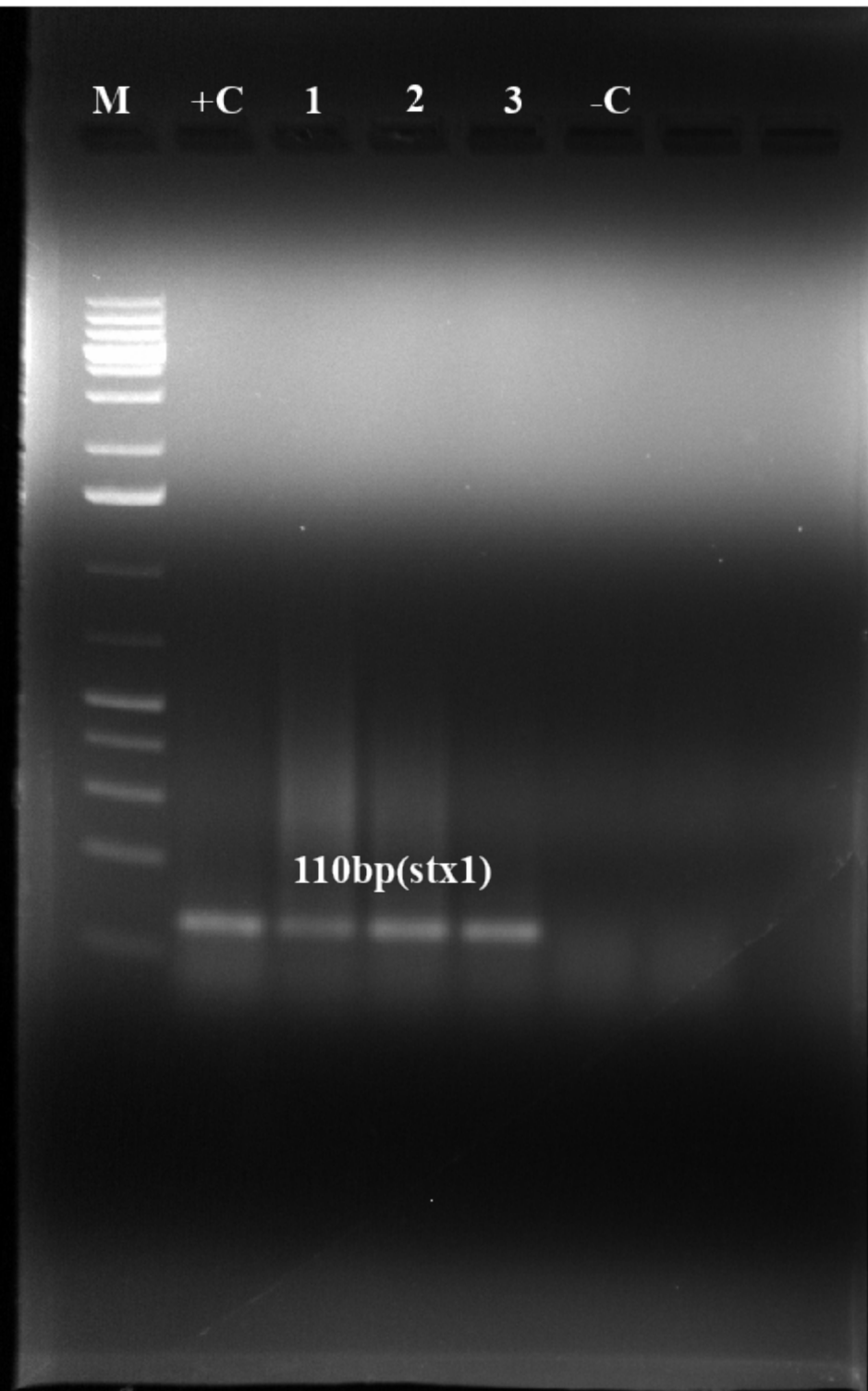

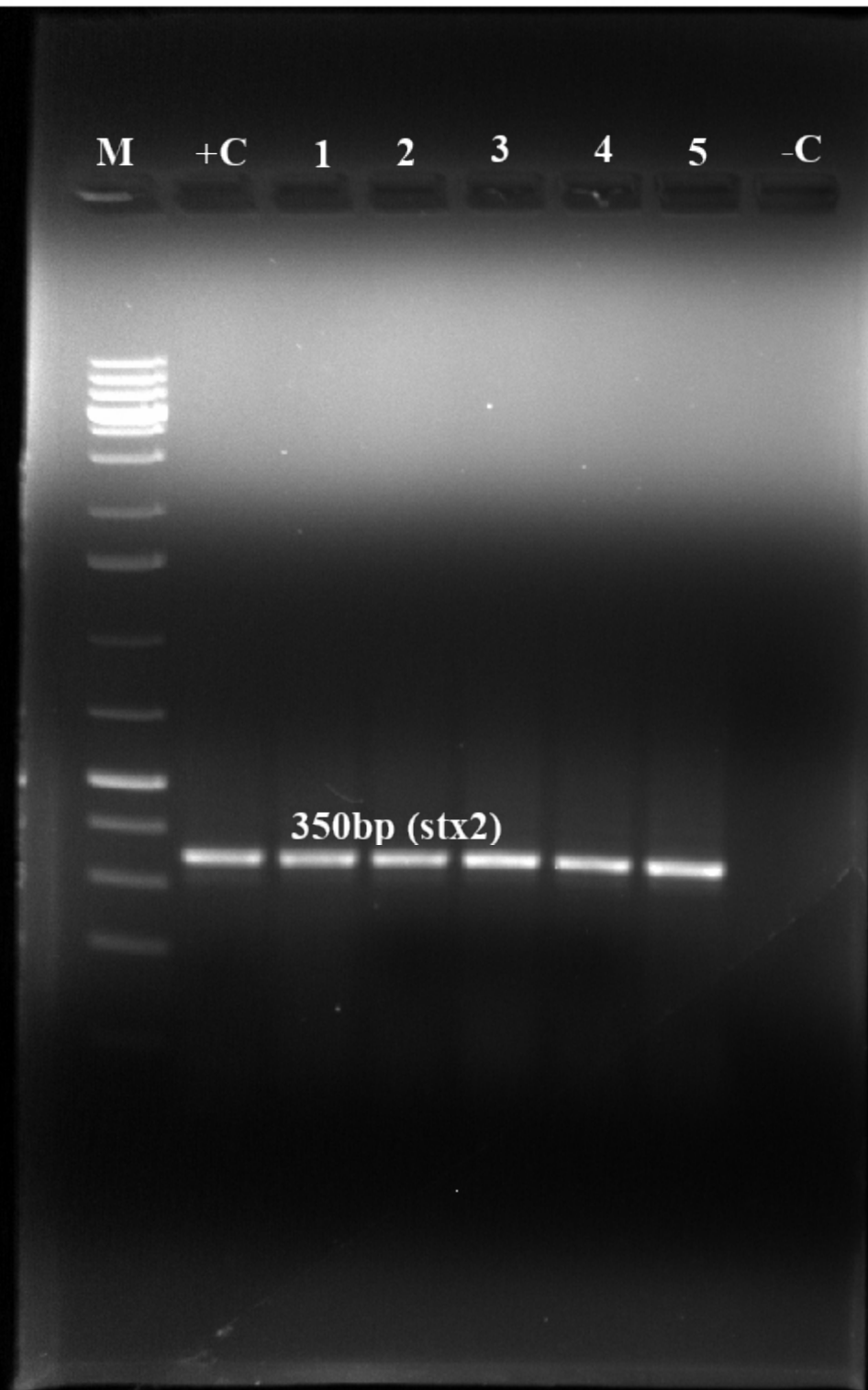

M +C 1 2 3 4 -C

167 bp(hlya)

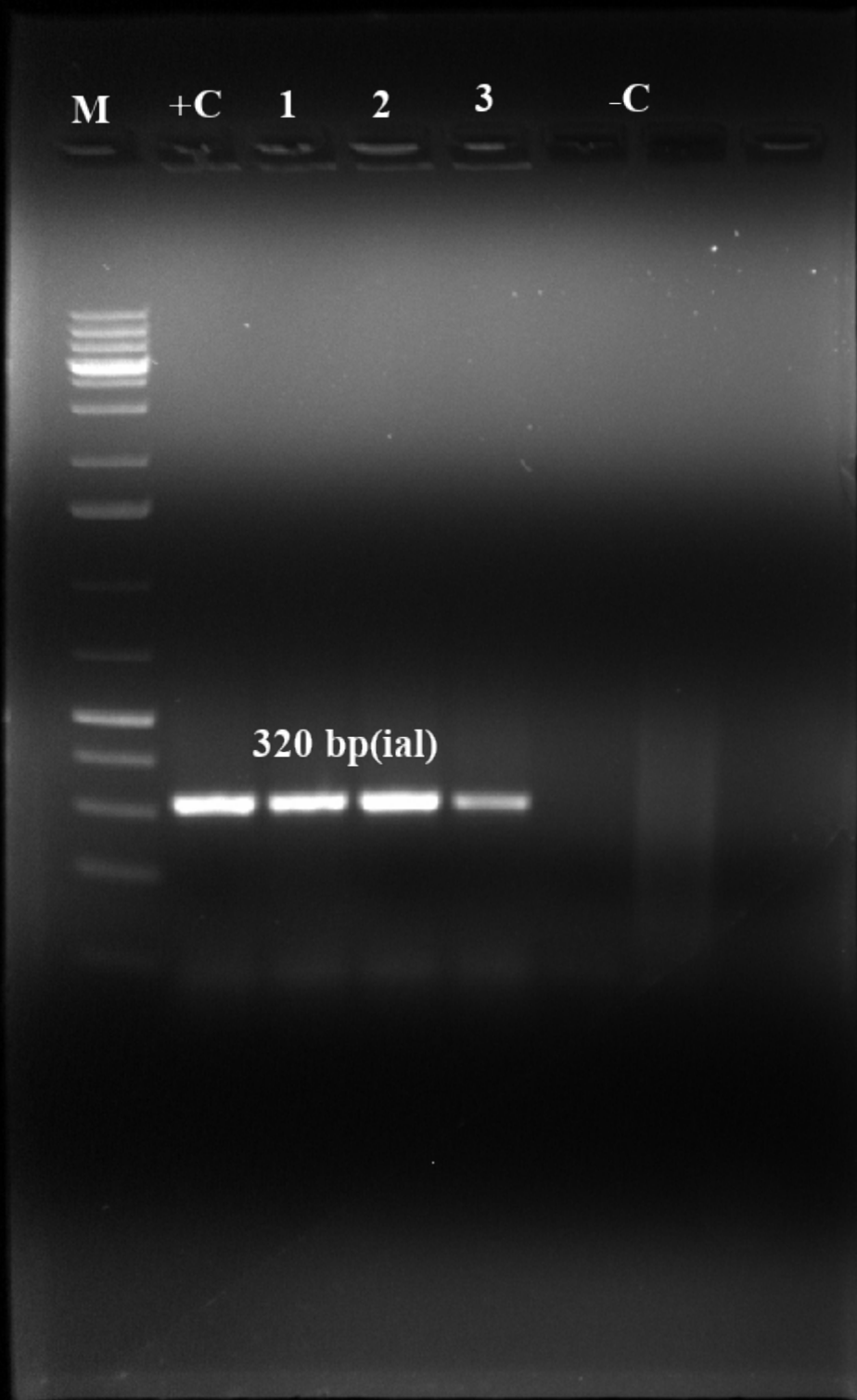

M +C 1 2 3 -C

630 bp(aatA)

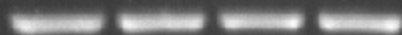

M +C 1 2 3 4 5 -c

294 bp(st)

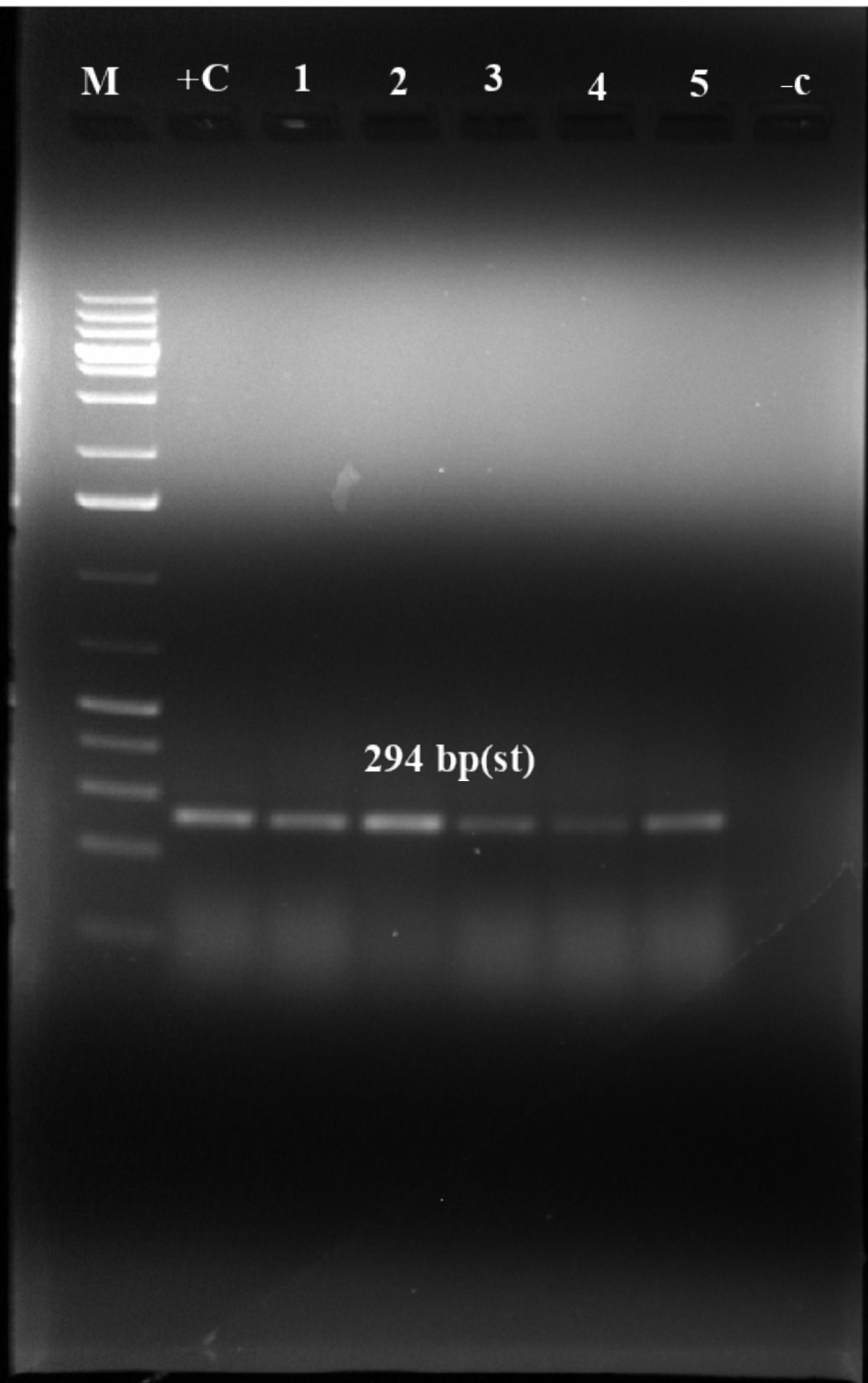

M +C 1 2 3 4 -C

696 bp(lt)

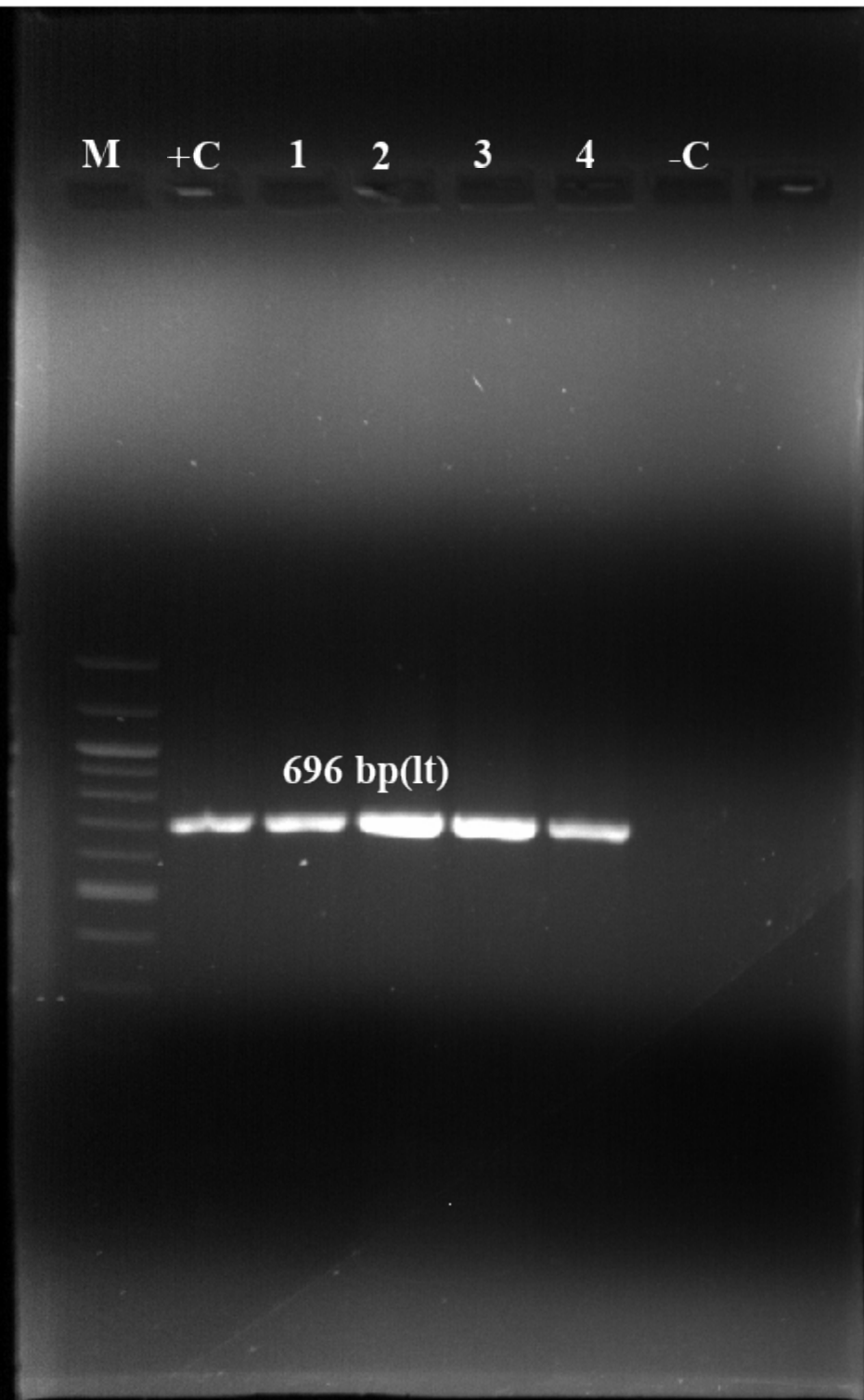

Supplement: S1 File — (PDF) [file pone.0275229.s003.pdf]
